# Dysautonomia and Implications for Anosmia in Long COVID-19 Disease

**DOI:** 10.3390/jcm10235514

**Published:** 2021-11-25

**Authors:** Alexandre Vallée

**Affiliations:** Department of Clinical Research and Innovation, Foch Hospital, 92150 Suresnes, France; alexandre.g.vallee@gmail.com

**Keywords:** COVID-19, dysautonomia, long COVID-19, anosmia, ACE2, neurotropism

## Abstract

Long COVID-19 patients often reported anosmia as one of the predominant persisting symptoms. Recent findings have shown that anosmia is associated with neurological dysregulations. However, the involvement of the autonomic nervous system (ANS), which can aggregate all the long COVID-19 neurological symptoms, including anosmia, has not received much attention in the literature. Dysautonomia is characterized by the failure of the activities of components in the ANS. Long COVID-19 anosmia fatigue could result from damage to olfactory sensory neurons, leading to an augmentation in the resistance to cerebrospinal fluid outflow by the cribriform plate, and further causing congestion of the glymphatic system with subsequent toxic build-up in the brain. Studies have shown that anosmia was an important neurologic symptom described in long COVID-19 in association with potential COVID-19 neurotropism. SARS-CoV-2 can either travel via peripheral blood vessels causing endothelial dysfunction, triggering coagulation cascade and multiple organ dysfunction, or reach the systemic circulation and take a different route to the blood–brain barrier, damaging the blood–brain barrier and leading to neuroinflammation and neuronal excitotoxicity. SARS-CoV-2 entry via the olfactory epithelium and the increase in the expression of TMPRSS2 with ACE2 facilitates SARS-CoV-2 neurotropism and then dysautonomia in long COVID-19 patients. Due to this effect, patients with anosmia persisting 3 months after COVID-19 diagnosis showed extensive destruction of the olfactory epithelium. Persistent anosmia observed among long COVID-19 patients may be involved by a cascade of effects generated by dysautonomia leading to ACE2 antibodies enhancing a persistent immune activation.

## 1. Introduction

Increasing numbers of patients convalescent with SARS-CoV-2 have complained of symptoms months after acute infection and have not returned to their initial health state prior to infection, leading us to suspect a long COVID-19 disease [1]. The observed symptoms are associated with disability, debilitating fatigue, breathlessness, headaches, muscle and/or joint pain, brain fog, memory loss, sensation of pressure on the chest, palpitations, nausea, anosmia, dramatic mood swings in combination with exercise intolerance and a relapsing-remitting pattern of recurrence [2]. Anosmia is commonly observed in mild as well as more severe COVID-19 [3,4,5,6]. Recent studies have shown that anosmia was one of the main symptoms observed in long COVID patients [7]. Anosmia presents spontaneous improvement over a two- or three-week period during acute COVID-19 infection. However, many COVID-19 patients remain anosmic for longer duration periods [8]. Moreover, long COVID-19 patients with or without hospitalization often reported anosmia as one of the predominant persisting symptoms [7,9,10,11].

Recent findings have shown that anosmia is associated with neurological dysregulations [12]. Indeed, the main common symptoms attributable to central nervous system (CNS) involvement include headache, anosmia, and dysgeusia [5,6]. Anosmia could be a result of viral interaction with non-neuronal cells of the mucosal surfaces in the olfactory epithelium and olfactory bulb [13,14]. However, the involvement of the autonomic nervous system (ANS), which can aggregate all these neurological symptoms (including anosmia) in long COVID-19 infection, has not received much attention in the literature [15]. Currently, recent studies have shown that these symptoms are the manifestation of a dysautonomia in long COVID-19 patients [16]. Here, we review evidence suggesting that dysautonomia could be responsible for anosmia occurring in long COVID-19 infection.

## 2. Dysautonomia in Long COVID-19 Patients

Dysautonomia is characterized by either the failure or the increased activities of the sympathetic or parasympathetic components in the ANS. Dysautonomia presents several clinical symptoms, such as fatigue, anosmia, orthostatic hypotension, dysfunction in heart rate variability, appearance of impotence, dysfunction of bladder, and damage to bowel functions. Dysautonomia could be acute or chronic, as well as progressive but also reversible and is associated with many pathologies, including alcoholism, diabetes, or Parkinson’s disease. Dysautonomia is also associated with viral infections, including hepatitis C virus, HIV, or Epstein–Barr virus [15]. Several etiologies can explain dysautonomia in long COVID-19 patients, such as neurotropism, hypoxia, and inflammation [9]. Nevertheless, it remains unclear whether dysautonomia associated with long COVID-19 directly results from the autonomic-virus pathway or post-infectious immune-mediated processes.

## 3. SARS-CoV-2, Blood–Brain Barrier and Anosmia

SARS-CoV-2 can lead to neuronal damages by affecting the nervous system as anosmia and ageusia were among the predominant persisting neurological symptoms described [17]. Indeed, similarly to other zoonotic coronaviruses, including Middle East respiratory syndrome-related coronavirus and SARS-CoV-1, SARS-CoV-2 possesses the capacity for neurological virulence [18]. COVID-19 infection may be responsible for invasion of the central nervous system (CNS) by a direct hematogenous and neural propagation [19]. SARS-CoV-2 in the airways may pass by the epithelial barrier invading blood and lymph circulation and then propagating towards the CNS. The diameter of the SARS-CoV-2 virus is 60 to 140 nm, which allows it to translocate through the blood–brain barrier (BBB) and gain entry into the CNS [20]. However, the neurobiological mechanisms of SARS-CoV-2 remain unclear despite the high prevalence of observed neurological complications being documented [21,22]. The processes contributing to neurological symptoms may include toxic or metabolic complications to respiratory disease, as well as consequences of the anti-SARS-CoV-2 immune response, such as cytokine release syndrome and excessive immune activation [23].

SARS-CoV-2 gains entry to the CNS by penetrating the BBB. Several possible pathways may explain this phenomenon: invasion and infection by vascular endothelial cells and invasion and infection by pericytes embedded in endothelial membrane [24]. Moreover, SARS-CoV-2 can infect macrophages and monocytes recruited across the BBB [25]. In parallel, SARS-CoV-2 can infect vascular endothelial cells to invade the BBB [26]. The S protein on SARS-CoV-2 can disrupt the BBB by interacting with brain endothelial cells and leading to cell damages and a reduction in BBB integrity [27]. The possible mechanisms of SARS-CoV-2 invading the CNS may explain the hallmarks of COVID-19 infection, such as anosmia and ageusia, which manifest the viral entry into the CNS [28].

## 4. Anosmia and Dysautonomia

One of the main explanations is that a larger area of the olfactory epithelium is damaged, with a more profound epithelium destruction including death of numerous olfactory receptor neurons [29]. Long COVID-19 anosmia fatigue could result from damage to olfactory sensory neurons, leading to an augmentation in the resistance to cerebrospinal fluid outflow by the cribriform plate, and further causing congestion of the glymphatic system with subsequent toxic build-up in the brain [30].

Recent studies have shown that anosmia was an important neurologic symptom described in long COVID-19 in association with possible COVID-19 neurotropism [9,31]. The dissemination throughout olfactory nerve projection by the infection of the nasal mucosa and sustentacular cells is a possible process of COVID-19 neuropathogenesis. Moreover, in the olfactory bulb of post-mortem COVID-19 patients, viral RNA has been observed [32].

The glossopharyngeal afferents, innerving the carotid body and the vagal afferents and implicated in the respiratory tract, have a major role in the monitoring of the organelle process and in controlling homeostasis through the activation of the ANS. Theses neurons are the primary sensory inputs of several reflex loops controlling many key functions, including heart rate, blood pressure, and airway caliber [33]. Pulmonary receptors expressed on afferent vagal nerve terminals in the lung present mechanical or chemical stimuli, translocating in the brainstem by small-diameter myelinated (Aδ) or unmyelinated (C)-fiber nerve axons. Vagal C-fiber afferents innervate the larynx response to laryngeal discomfort whereas the afferent information arrives from the vagal and glossopharyngeal nerves to merge at the nucleus of the tractus solitarius (NTS), a major site of critical homeostasis signaling [33]. The biological phenomenon underlying the anosmia in COVID-19 patients remains unclear. However, the observed disassociation may be observed in patients showing lesions in the glossopharyngeal or vagus nerves due to cranial nerve affections [34]. Dysautonomia may enhance afferent baroreflex failure [9]. This mechanism generates numerous damages at the afferent baroreceptor pathway, starting from baroreceptors in carotid bodies to the vagal and glossopharyngeal nerve fibers, and then progressing to the NTS [15].

## 5. ACE2 Hypothesis in Long COVID-19

The SARS-CoV-2 virus contains a simple strain RNA as genetic material and is composed of three protein structures: the spike, the envelope, and the membrane. The spike binds to the Angiotensin-Converting Enzyme 2 receptor (ACE2) and both the envelope and the membrane implicate the genetic material [35]. Several studies have observed that there is a genetic variation in the human receptor for the virus, ACE2, in the European population, increasing this receptor expression. Europeans could be more predisposed to anosmia in comparison to Asians, precisely because they may mainly have receptors for the entry of the SARS-CoV-2 virus [36,37].

Moreover, the SARS-CoV-2 virus invades cells through ACE2 in association with TMPRSS2 (a protease-mediating S-protein cleavage). The main targets of SARS-CoV-2 are non-neuronal cells. Furthermore, the average recovery of smell is two weeks, a time span not compatible with the regeneration of neuronal cells [38,39]. Soluble platelet-derived growth factor receptor β (sPDGFRβ) has been proposed as a new candidate biomarker of blood–brain barrier function [40,41,42]. sPDGFRβ is mainly expressed in brain pericytes [43], a cell type with ACE2 expression as well as the viral cofactor TMPRSS2, making pericytes a possible target for COVID-19 infection [44].

ACE2 is the viral receptor for the SARS-CoV-2 virus and is expressed as both a membrane-bound form and a soluble form. The biological role of ACE2 is to transform the octapeptide angiotensin II (Ang II) to angiotensin (1–7). Ang II binds to AT1 receptor to generate an immune stimulation [45,46]. Ang (1–7) binds to the Mas receptor to inhibit the process of inflammation [47]. The increased levels of ACE2 protein are associated with decreased effects modulated by the stimulation of the AT1 receptor including immune stimulation (i.e., augmented ACE2 activity leads to inflammation inhibition). The link between SARS-CoV-2 and ACE2 leads to a decrease in the activity of this enzyme [48,49]. The immune system is enhanced in long COVID-19 infection. For instance, antinuclear [50], antiphospholipid [51] and anti-interferon [52] antibodies have been found after infection [53]. Antibodies against ACE2 may downregulate the activity of both soluble and membrane-bound ACE2 activating the receptors for Ang II and stimulating the immune system [53,54,55]. These recent findings showed that the ACE2 antibodies in the plasma could decrease the activity of ACE2. This downregulation could mainly damage the ACE2 enzyme that is tissue-bound as well as the activity of soluble ACE2. This provides a possible process for damage to the balance of angiotensin peptides to increase Ang II and to activate the immune system. Thus, two pathways of evidence can support the hypothesis that anti-idiotypic antibodies can enhance long COVID-19 symptoms [53,54,56].

## 6. ACE2 and Dysautonomia in Long COVID-19

ACE2 was observed in neurons of the brain [57] as neuropilin-1 receptor (NRP-1) was found in both the olfactory system [58] and neurons of the olfactory epithelium [59]. The ACE2 receptors are expressed in the brain and glial cells and SARS-CoV-2 acts via neuronal as well as non-neuronal pathways [60]. Several studies have suggested that the SARS-CoV-2 virus enters via the olfactory epithelium and affects the expression of both TMPRSS2 and ACE2 to facilitate SARS-CoV-2 neurotropism [13,61]. Moreover, SARS-CoV-2 exhibits neuroinvasive capacity in an ACE2-dependent manner to induce neuronal death [57] leading to the initiation of a neurotropism in these patients [62,63]. Other studies have shown that SARS-CoV-2 can damage the integrity of choroid plexus epithelium in the brain organoids of hippocampal-like regions [64]. Moreover, a second possible route of brain invasion by the SARS-CoV-2 virus could be a viral entry mediated by both ACE2 and NRP-1 [59,65]. Thus, the SARS-CoV-2 virus can either travel via peripheral blood vessels causing endothelial dysfunction, triggering coagulation cascade and multiple organ dysfunction, or reach the systemic circulation and take a different route to the blood–brain barrier, damaging the blood–brain barrier leading to neuroinflammation and neuronal excitotoxicity [66,67]. COVID-19 neural invasion via the peripheral nervous system nerve terminal leads to viral replication and retrograde transportation to soma leading to invasion of the central nervous system, and subsequent neurological manifestations for a long period post infection [60].

## 7. ACE2, Anosmia and Long COVID-19

Recent studies have shown an association between the expression of ACE2 receptor and age [68]. ACE2 receptors become more prevalent in adults than in children [37,61], explaining that young people exhibit a less severe prognosis [69,70].

Moreover, women are more likely to develop olfactory disorders than men in COVID-19 disease [5,70,71]. A possible explanation could be that incomplete X chromosome inhibition may contribute to overexpression of ACE2 [29]. A recent study has shown that anosmia, one of the main symptoms of long COVID-19, was more likely increased with aging and female gender [11]. Chen et al., observed that ACE2 immunohistochemical expression was 200 to 700 times greater in the sustentacular cells of the olfactory neuroepithelium than it was in nasal or tracheal epithelia [66]. Another study showed high levels of expression of both ACE2 and TMPRSS2 on the sustentacular cells of the olfactory epithelium [13]. This previous study has shown the absence of ACE2 expression on olfactory sensory neurons [13]. A post-mortem study of two patients with anosmia presented focal atrophy of the olfactory epithelium, leukocytic infiltration of the lamina propria and evidence of axonal damage in the olfactory nerve fibers [72]. Patients with anosmia persisting 3 months after COVID-19 diagnosis showed extensive destruction of the olfactory epithelium [73].

Another possible pathway could be the interaction between ACE2 receptor and adipose tissue in obese COVID-19 patients [74,75]. ACE2 is highly expressed in adipose tissue, especially in visceral fat, suggesting an essential role for this tissue in determining COVID-19 disease severity and duration [76,77]. However, this possible link remains unclear in long COVID-19 patients and should be investigated.

## 8. Conclusions

Persistent anosmia observed among long COVID-19 patients may be involved via a cascade of effects generated by dysautonomia leading to ACE2 antibodies enhancing a persistent immune activation.

## Data Availability

Not applicable.
